# Association between maternal cancer and the incidence of cancer in offspring

**DOI:** 10.1007/s10654-025-01206-z

**Published:** 2025-02-17

**Authors:** Su-Min Jeong, Jihye Heo, Kyujin Choi, Park Taegyun, Soo-Young Oh, Jonghan Yu, Danbee Kang

**Affiliations:** 1 Department of Family Medicine, Seoul National University Hospital, Seoul, Republic of Korea; 2 Department of Medicine, Seoul National University College of Medicine, Seoul, Republic of Korea; 3 Department of Clinical Research Design and Evaluation, Samsung Advanced Institute for Health Science and Technology, Sungkyunkwan University, Seoul, Republic of Korea; 4 Center for Clinical Epidemiology, Samsung Medical Center, Sungkyunkwan University School of Medicine, Seoul, Republic of Korea; 5 National Health Insurance Service, Wonju, Republic of Korea; 6 Department of Obstetrics and Gynecology, Samsung Medical Center, Sungkyunkwan University School of Medicine, Seoul, Republic of Korea; 7 Department of Surgery, Samsung Medical Center, Sungkyunkwan University School of Medicine, Seoul, Republic of Korea

**Keywords:** Female cancer survivors, Offsprings cancer, Maternal factors, Childhood outcomes

## Abstract

Despite the growing population of young cancer survivors of reproductive age, the risk of cancer in offspring born to female cancer survivors has yielded inconsistent results. Therefore, this study aimed to investigate the risk of cancer among the offspring of female cancer survivors by maternal age at delivery, maternal age at cancer diagnosis, maternal cancer type, and the time interval between cancer diagnosis and pregnancy. Using nationwide retrospective mother–child linked data from the Korean National Health Insurance Service, we included the first child (N = 8031) of female cancer survivors aged < 40 years after excluding thyroid cancer survivors and matched controls (N = 24,093) between 2005 and 2019. Subgroup analysis was performed according to maternal age at delivery, maternal age at cancer diagnosis, maternal cancer type, and the interval between cancer diagnosis and delivery. Among the offspring, 19 children of cancer survivors and 30 in the control group were diagnosed with cancer, with a mean age of 2.0 years at diagnosis. The most prevalent cancer type was leukemia (26.5%), followed by liver tumor (10.2%) and brain tumor (8.2%). The hazard ratio (HR) for cancer in the offspring of female cancer survivors was 1.91 (95% confidence interval (CI) = 1.07–3.38) demonstrating consistently high risk over the follow-up period. HRs for cancer risk in offspring were high across all subgroups despite the low statistical power. Our study indicated that offspring born to maternal cancer survivors had an increased risk of cancer.

**Supplementary Information:**

The online version contains supplementary material available at 10.1007/s10654-025-01206-z.

## Background

As the incidence of early-onset cancer (< 50 years) has sharply increased worldwide, a notable increase in the number of females diagnosed with cancer during their reproductive period has been observed [1]. As cancer survival rates have improved, the growing population of survivors of reproductive age has raised interest in infertility and the potential transgenerational effects of cancer treatment, even after successful pregnancy. Several studies have reported adverse birth outcomes, such as preterm birth, low birth weight, and birth defects, among offspring born to survivors of maternal cancer [2–5]. However, few studies have focused on the long-term health outcomes of offspring born to maternal cancer survivors, such as cancer risk [3, 6–8]. Young female cancer survivors had concerns about whether their offspring may have an increased risk of cancer, as cancer treatment was believed to have the potential to cause DNA damage and mutation, even in normal reproductive cells [9]. According to the survey, 63.3% of young female cancer survivors expressed concerns about passing on the cancer risk to their children [10]. Furthermore, the fear that their children would develop cancer was one reason why cancer survivors chose not to have children [11].

Inconsistent findings regarding the risk of cancer in offspring born to female cancer survivors have been reported in previous studies. A large population-based Danish study reported a 2.2-fold increased risk of cancer in offspring exposed to maternal cancer in utero [6]. A Korean study involving 15,221 children of mothers with cancer demonstrated non-significant differences in cancer risk compared with that in the control group (hazard ratio [HR] = 0.98, 95% confidence interval [CI] = 0.64–1.51) [3]. Given that the risk of cancer was assessed as a long-term health outcome, its evaluation was somewhat limited [3, 6]. A previous study of 9,877 Finnish children born after their parents’ cancer diagnosis identified an increased cancer risk (standardized incidence ratio [SIR] = 1.67, 95% CI 1.29–2.12) [7]. After excluding those with hereditary cancer syndromes, the increased risk of cancer disappeared (SIR = 1.03, 95% CI 0.74–1.04). This study included heterogeneous cancer survivors encompassing both parents (father and mother) and those diagnosed with cancer across a broad range of ages from 0 to 34 years. This diverse study population may pose challenges for consistent interpretation. A Nordic collaborative study reported similar results, with no significant evidence of an increased risk of sporadic cancer among the offspring of childhood cancer survivors [8]. Including only childhood cancer survivors may not fully reflect the effect of cancer treatment during reproductive age on the risk of cancer among the offspring of female cancer survivors.

Therefore, this nationwide population-based cohort study aimed to investigate the risk of cancer among the offspring of female cancer survivors, focusing on those of reproductive age. In addition, we performed a subgroup analysis according to maternal age at delivery, maternal age at cancer diagnosis, maternal cancer type, and the time interval between cancer diagnosis and pregnancy to enhance our understanding of the risk of cancer among the offspring.

## Methods

### Data source and study cohort

We conducted a nationwide retrospective cohort study using the Korean National Health Insurance Service (K-NHIS) database, which represents the entire population of South Korea. The database comprises the national records of all covered inpatient and outpatient visits, procedures, and prescriptions from 2004 to 2020 [12]. To improve the causal relationship, we mimicked a targeted trial [13]. We specified our research question, target population, exposure, comparators, outcomes, and timing according to the principles of a randomized controlled trial (Supplementary Table 1).

**Fig. 1:**
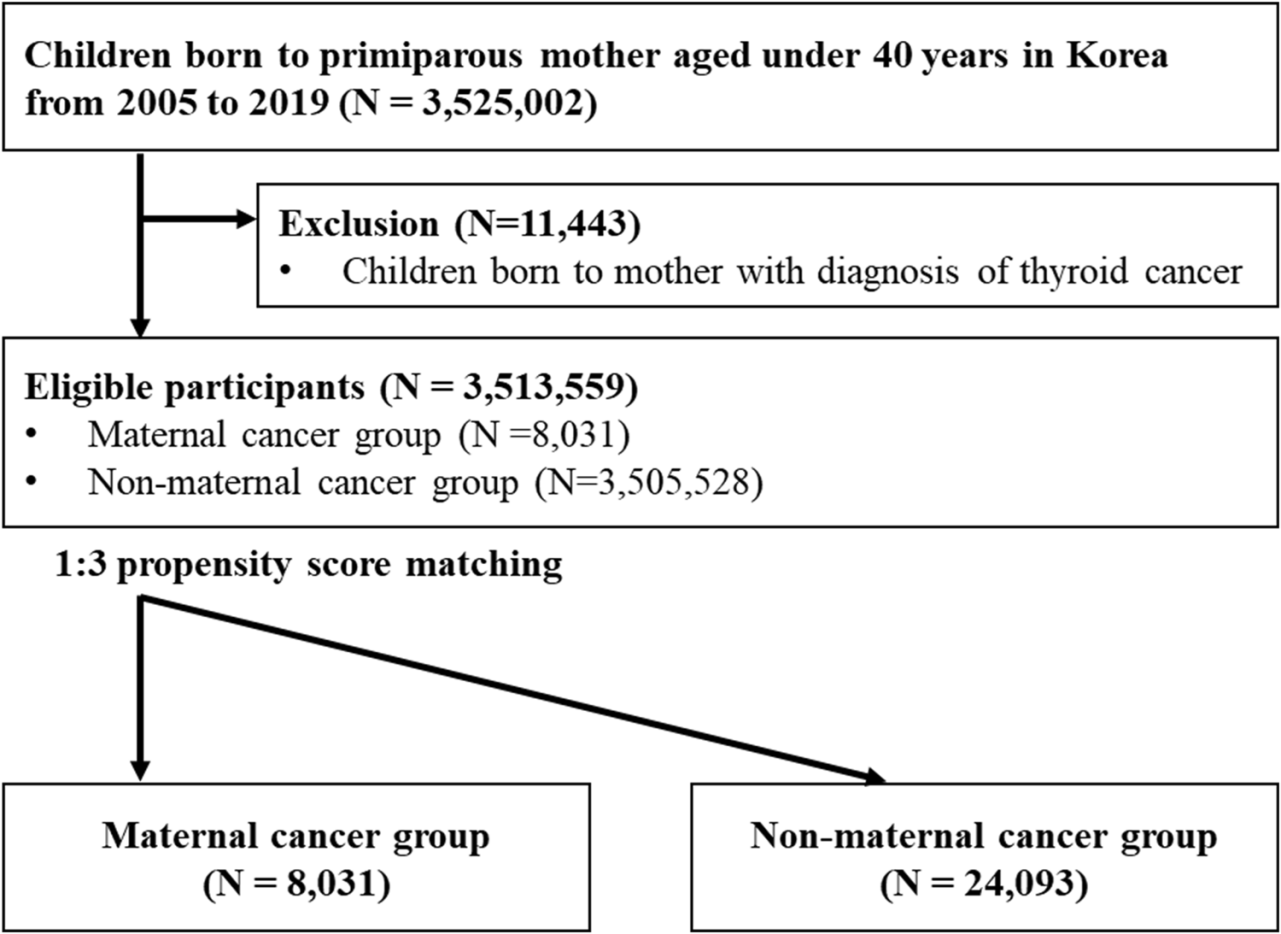
Flowchart of the study participants. Matching variables were age at delivery, delivery date, income, residential area, history of abortion, history of stillbirth

Our cohort included all live births between January 1, 2005, and December 31, 2019, with a washout period of 2004 and a follow-up period of 2020. The NHIS links all claims data of mothers with the claims data of their offspring. We limited our study to children born to females under 40 years of age who gave birth to their first child, recognizing that maternal age can significantly affect pregnancy outcomes (N = 3,525,002). Of eligible participants, 19,474 were born to mothers with cancer. Due to concerns regarding overdiagnosis issues with thyroid cancer [14], we further excluded children born to mothers with thyroid cancer. Finally, 8031 mothers with cancer were included in the study. Controls were matched with mothers who had cancer at a ratio of 1:3 using a propensity score (PS). This study included 8,031 children born to mothers with cancer and 24,093 controls **(**Fig. 1**).**

The requirement for informed consent was waived as this study was conducted using anonymized claims data. This study was approved by the Institutional Review Board of Samsung Medical Center, South Korea (SMC 2021-08-107).

### Measurement

The K-NHIS data comprise individual-level demographics and all records of diagnosis and healthcare utilization (e.g., drug prescriptions and medical procedures) provided during inpatient, outpatient, and emergency department visits. Moreover, NHIS claims for inpatient and outpatient visits, procedures, and prescriptions were coded using the 10th revision of the International Statistical Classification of Diseases (ICD) [15].

A cancer diagnosis was defined as the presence of the same C code more than three times within a year or an inpatient hospitalization with the C code [16]. In Korea, once a person receives a cancer diagnosis, they are registered with the National Cancer Registry with a specific code (V193). This indicates that the individual has been diagnosed with cancer and is receiving special insurance benefits. Once a person receives V193, the code becomes a part of the medical records and subsequent claims. Cancer diagnosis during pregnancy was defined as the date of diagnosis, ranging from the last menstrual period to the date of delivery. The last menstrual period was estimated using a previously validated algorithm to estimate the gestational age in administrative healthcare databases [17]. The type of cancer was identified by the ICD-10 code at the visit based on the cancer definition (Supplementary Table 2). The definition of cancer incidence in offspring was identical to that used for mothers.

We considered a broad range of covariates as potential confounders or proxies for potential confounders: maternal age, income, residential area at delivery, and maternal comorbidities, including history of congestive heart failure, history of abortion and stillbirth, and comorbidities during pregnancy. Data on age and income at the time of the first screening were obtained from an insurance eligibility database. Income level was categorized into percentile groups ( ≤ 30th, > 30th to ≤ 70th, and > 70th percentiles). Residential areas at the time of the first screening examination were classified as either metropolitan or rural. Metropolitan areas were defined as Seoul, six metropolitan cities, and 15 cities with populations > 500,000 officially designated as municipal cities (http://www.mois.go.kr). Maternal comorbidities within the year before birth were defined using ICD-10 codes, including hypertension and diabetes. We also identified adverse events during pregnancy, including hypertensive disorders (ICD-10 codes O14, O11, O15, O13, O16, I10, and O10), gestational diabetes (ICD-10 codes O244), and overt diabetes (ICD-10 codes O240, O241, O242, O243, E10, E11, E12, E13, and E14). Preterm birth was defined as the presence of ICD-10 codes O601, O603, P072, O073, or P590.

### Statistical analysis

For 1:3 matching, a propensity score (PS) was generated using birth date, income, residential area, maternal age at delivery, history of abortion, and history of stillbirth. PS matching was performed to minimize the potential impact of confounders on the exposure outcomes. Matching was performed using a greedy algorithm (caliper = 0.1).

The incidence of cancer in the offspring was tracked from the date of birth until the date of cancer diagnosis, death, or the end of follow-up (December 31, 2020), whichever occurred first. Incidence percent per year was calculated as the number of events per 100 person-years of follow-up. The cumulative incidence of each maternal cancer outcome was evaluated using Kaplan–Meier curves. Hazard ratios (HRs) and the corresponding 95% confidence intervals (CIs) were calculated using Cox proportional hazards models. The proportionality of the hazards was assessed by visual inspection of log-minus log plots and Schoenfeld residuals.

Due to lack of power, exploratory subgroup analysis was performed according to maternal age at delivery (< 30, 30–34, and 35–39 years). In addition, we also performed exploratory stratified analysis by characteristics of maternal cancer including maternal age at cancer diagnosis (9–19, 20–24, 25–29, 30–34 and 35–39), maternal cancer type (non-Hodgkin lymphoma [NHL], colorectal, cervix, breast and ovary), and time between cancer diagnosis and pregnancy (diagnosed during pregnancy, < 1, 1 to < 3, 3 to < 5 and ≥ 5).

All analyses were two-sided, and *P*-values < 0.05 were considered statistically significant. Statistical analyses were performed using SAS version 9.2 (SAS Institute Inc., Cary, NC, USA) and R software version 3.3.2 (Free Software Foundation Inc., Boston, MA, USA).

## Results

### Baseline characteristics

After matching, all standardized mean differences (SMDs) of the difference between the control and cancer survivors were less than 0.2 (Table 1). The mean ages of the cancer survivors and those in the control group (non-cancer) were 33.4 and 33.5 years, respectively. Cancer survivors were likely to have comorbidities such as hypertension, and diabetes mellitus. Cancer survivors had more cesarean deliveries (51.8%) and preterm births (10.0%) than those in the control group. The mean maternal age at cancer diagnosis was 29.2 (standardized deviation, 4.5), with breast cancer (18.4%) being the most prevalent cancer type, followed by ovarian (17.4%) and cervical (12.9%) cancers. As displayed in Supplement Table 3, 19 and 30 children were diagnosed with cancer in the cancer survivor and control groups, respectively. The mean age at cancer diagnosis among offspring was 2.0 years, with children born to cancer survivors being diagnosed at a younger age than those in the control group. The most frequent cancer was leukemia (26.5%), followed by liver tumors (10.2%), and brain tumors (8.2%).

**Table 1:** Characteristics of study participants by maternal cancer

	Non-cancer (N = 24,093)	Cancer (N = 8031)	SMD
Characteristics of mothers			
Maternal age (years), mean (SD)	33.5 (3.35)	33.4 (3.45)	0.035
Income level			
Q1 (lowest)	194 (0.8)	88 (1.1)	0.030
Q2	4270 (17.7)	1598 (19.9)	0.056
Q3	12,245 (50.8)	3792 (47.2)	0.072
Q4. (highest)	6988 (29.0)	2355 (29.3)	0.007
Unknown	396 (1.6)	198 (2.5)	0.058
Rural areas	6463 (26.8)	2355 (29.3)	0.056
Comorbidities			
Hypertension	193 (0.8)	121 (1.5)	0.066
Diabetes Mellitus	286 (1.2)	204 (2.5)	0.100
History of adverse pregnancy outcomes			
History of abortion	5548 (23.0)	2006 (25.0)	0.046
History of stillbirth	107 (0.4)	54 (0.7)	0.031
Comorbidities during pregnancy			
Hypertensive disorder	953 (4.0)	343 (4.3)	0.016
Diabetes mellitus	7752 (32.2)	2727 (34.0)	0.027
Birth events			
Cesarean delivery	11,159 (46.3)	4160 (51.8)	0.110
Pre-term birth	1306 (5.4)	807 (10.0)	0.174
Maternal cancer characteristics			
Cancer diagnosis age (years), mean (SD)	–	29.2 (4.5)	
Type of cancer	–		
Breast	–	1474 (18.4)	
Ovary	–	1395 (17.4)	
Cervix	–	1034 (12.9)	
NHL	–	510 (6.4)	
Stomach	–	532 (6.6)	
Colon rectal	–	338 (4.2)	
Brain	–	264 (3.3)	
Uteri	–	291 (3.6)	
Others	–	2193 (27.3)	

SMD, Standardized mean difference; GA, Gestational age; NHL, Non-Hodgkin lymphoma; Values are expressed as mean ± SD or N (%)

### Incidence of cancer among offspring

**Fig. 2:**
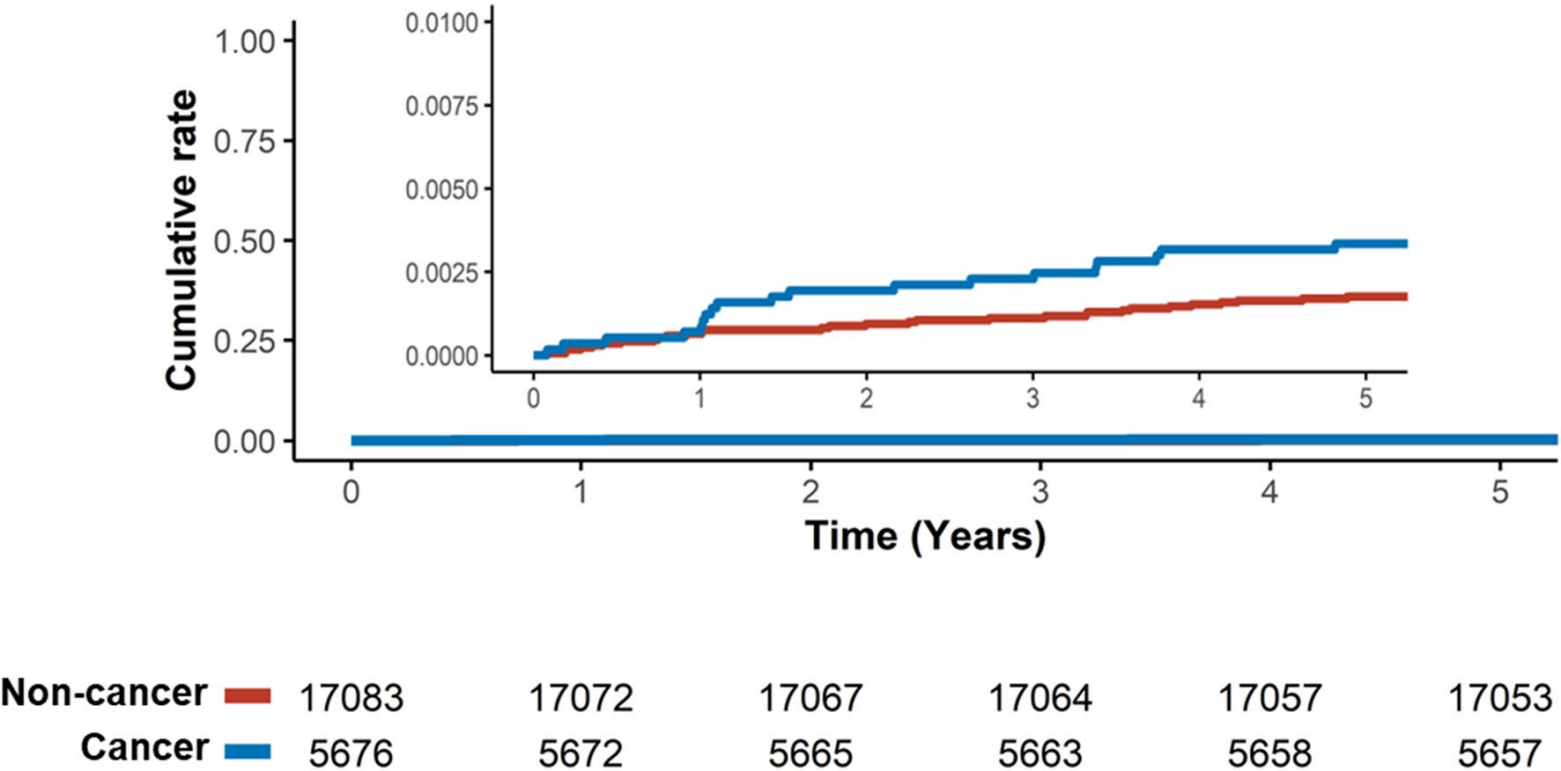
Kaplan–Meier curve for cancer incidence among offspring according to maternal cancer

The incidence of cancer in the offspring was consistently higher particularly in children born to cancer survivors than that in controls over time (Fig. 2). The HR for cancer in offspring born to cancer survivors was 1.91 (95% CI 1.07–3.38) **(**Table 2**).** Despite the lack of statistical significance, the HRs for the risk of cancer in the offspring were consistently higher across all subgroups of maternal age at delivery.

**Table 2:** Cancer risk among offspring of cancer survivors comparing with non-maternal cancer

	Number of events (incidence rate % a years)		Hazard ratio (95% CI)
	Non-cancer	Cancer	
All population			
Overall	30 (0.03)	19 (0.05)	**1.91 (1.07, 3.38)**
Maternal age at delivery, year			
< 30	1 (0.01)	2 (0.04)	5.43 (0.49, 59.84)
30 to 34	15 (0.03)	7 (0.04)	1.43 (0.58, 3.50)
35 to 39	14 (0.03)	10 (0.07)	2.18 (0.97, 4.91)

CI, confidence interval

In an additional subgroup analysis, the risk of cancer in offspring born to cancer survivors was high regardless of maternal age at cancer diagnosis. **(**Table 3**)** Offspring of survivors with NHL (HR = 9.50; 95% CI 3.96–22.82) or colorectal cancer (HR = 5.55; 95% CI 1.32–23.29), and survivors who received chemotherapy and/or radiotherapy (HR = 2.75; 95% CI 1.30, 5.78) showed a potential increased risk of cancer. Similarly, although there appeared to be a trend in cancer risk among offspring based on the interval between maternal cancer diagnosis and pregnancy (HR = 1.46, 95% CI 0.20–10.72 during pregnancy; HR = 2.51, 95% CI 0.88–7.15 within 1 year of diagnosis), the results were not statistically significant.

**Table 3:** Subgroup analysis of cancer risk among offspring of cancer survivors comparing with control

	Hazard ratio (95% CI)
Maternal age at cancer diagnosis, year	
9–19	–
20–24	1.77 (0.42, 7.42)
25–29	2.00 (0.88, 4.55)
30–34	1.97 (0.90, 4.29)
35–39	1.99 (0.47, 8.31)
Maternal cancer type	
NHL	9.50 (3.96, 22.82)
Colorectal	5.55 (1.32, 23.29)
Cervix	2.35 (0.72, 7.69)
Breast	1.63 (0.50, 5.34)
Ovary	0.57 (0.08, 4.15)
Treatment modality	
Other treatments	1.49 (0.73, 3.06)
Chemotherapy or/and radiation therapy	2.75 (1.30, 5.78)
Time between maternal cancer diagnosis and pregnancy	
Diagnosed during pregnancy	1.46 (0.20, 10.72)
Survivor period before pregnancy, year	
< 1	2.51 (0.88, 7.15)
1 to < 3	1.61 (0.63, 4.15)
3 to < 5	1.46 (0.44, 4.77)
> 5	2.37 (0.99, 5.71)

CI, confidence interval; NHL, non-Hodgkin lymphoma; CNS, central nervous system

## Discussion

In this population-based retrospective cohort study, we identified a 1.91-fold increased risk of cancer among children born to survivors of maternal cancer compared with the risk in those in the control group. Cancer risk in the offspring was consistently higher than that in the control group over time. In the subgroup analysis, the association was consistently higher; in particular, offspring born to mothers of advanced age at birth (35–40 years) and survivors with NHL and colorectal cancer exhibited the highest risk of cancer.

To the best of our knowledge, our study is the first to comprehensively investigate cancer risk among offspring born to mothers who were cancer survivors, including a long-term follow-up. Few studies have reported inconsistent findings on long-term health outcomes in children born to survivors of maternal cancer. A Danish study compared the risk of cancer among children whose mothers were diagnosed with cancer during pregnancy with a reference group without maternal cancer and identified the risk of cancer to be elevated, with an HR of 2.2 (95% CI 1.0–4.9) [6]. However, this study adjusted only for calendar period and maternal attained education at birth without considering maternal and postnatal health status. Furthermore, only six cases of cancer were noted among the 666 offspring of cancer survivors, which should be interpreted with caution. A large Nordic study reported an increased standardized incidence ratio (SIR) for non-retinoblastoma cancer (SIR = 1.6, 95% CI 1.1–2.4), but the increase in SIR was not statistically significant when analyses were limited to sporadic cancers (SIR = 1.3, 95% CI 0.8–2.0) [8]. Another Finnish study demonstrated similar findings, indicating no increase in the risk of sporadic cancer among children of survivors of non-hereditary cancer after excluding those with hereditary cancer syndrome [7]. The different findings in our study could be partly attributed to the inclusion of hereditary cancers. Other studies on the risk of cancer in offspring were performed on childhood and adolescent cancer survivors in the 1980s. The studies included the offspring of both male and female cancer survivors and did not focus on cancer survivors during the reproductive period [18, 19]. However, the limited number of cancers observed in offspring restricts reliable risk estimation. In addition, these studies conducted interviews to identify the health of the offspring, potentially causing recall bias. Furthermore, since the previous study had limited results with a relatively short follow-up period [3], the risk of epithelial tumors, which tend to increase with age [20] may not have been accurately reflected. However, in our study, we discovered that the incidence of epithelial tumors in the offspring was higher than that in the offspring of women without cancer.

In our study, the risk of cancer was consistently elevated across the groups, particularly among those with maternal age at delivery under 30 years, despite the low incidence (one case vs. two cases). Additionally, maternal age at birth (35–40 years) may reinforce the cancer risk in offspring born to cancer survivors. Compared with children born to mothers aged 20–24 years, those born to mothers of older age had a 13–36% higher risk of pediatric cancer [21]. Maternal age > 35 years is associated with aneuploidy in the offspring [22], and such genetic defects could increase cancer risk [23]. Maternal age is correlated with the frequency of de novo germline mutations in offspring [24]. Furthermore, there might be have possible transgenerational effects of cancer treatment, even after a successful pregnancy. Among cancer survivors, patients who received chemotherapy or/and radiotherapy which could affect to the somatic mutation [25] were stronger effects on the cancer risk of offspring than other treatment compared to offspring of mothers without cancer. The majority of germline mutations are related to somatic mutations known as oncogenic mutations leading to various tumors [26].

We identified the highest risk of cancer in offspring born to survivors with NHL, followed by those with colorectal cancer. Common cancer types in the offspring were leukemia, liver tumors, and brain tumors. A previous study based on a small population (N = 382) reported no evidence of an increased risk of cancer among the offspring of survivors of childhood or adolescent leukemia and NHL [27]. Although NHL is regarded as a non-hereditary cancer, parental NHL confers an increased risk of NHL and other hematopoietic malignancies [28]. In addition to shared genetic predisposition, similar environmental exposures, lifestyle factors such as dietary patterns, and shared infection with the mother might play a role in the development of cancers in offspring [28]. Conversely, the health status of offspring born to colorectal cancer survivors is related to neonatal outcomes including 2.3-fold higher odds of preterm birth [29]. Adverse neonatal outcomes such as preterm birth could increase the risk of cancer, particularly for acute myeloid leukemia, retinoblastoma, and germ cell tumors (odds ratio = 1.28, 95% CI 1.06–1.57) [30].

We also explored the risk of cancer among the offspring according to the interval between maternal cancer diagnosis and pregnancy. With cautious interpretation due to statistical non-significance, the cancer risk of offspring born to cancer survivors consistently increased across all subgroup categories. However, the offspring of cancer survivors who were diagnosed during pregnancy (HR 1.46) and within 1 year after cancer diagnosis (HR 2.51) did not demonstrate a significantly increased risk. Additionally, cancer diagnosis during pregnancy and shortly before pregnancy is challenging for both the mother and the fetus. Offspring exposed to maternal cancer in utero and within 1 year may be vulnerable to cancer diagnosis, cancer treatment, and diagnostic procedures. Cancer associated with pregnancy involves metabolic disturbances toward catabolic phases, resulting in a severe reduction in the nutrient supply for fetal growth [31]. In addition, the mutagenic effects of cancer treatment on the reproductive organs shortly before and during pregnancy may increase the risk of childhood leukemia and other tumors in the offspring [32].

Currently, cancer survivorship care prioritizes addressing fertility and sexual dysfunction [33]. Beyond the health concerns of cancer survivors, the health status of offspring born to cancer survivors is also crucial for ensuring their overall quality of life. In this context, our study highlights the importance of incorporating the long-term health outcomes of offspring into cancer survivorship care to provide comprehensive support to cancer survivors and their families.

This study had several limitations. First, although we included important confounders, we acknowledge the possibility of residual confounding due to unmeasured factors such as genetic predisposition, family history of cancer and lifestyle factors. In this study, the E-value was 3.23, which means that an unmeasured confounder would have to have a risk ratio of at least 3.23 with both the maternal cancer and the offspring cancer to nullify our observed association [34]. However, since these unmeasured factors are already highly correlated with the current covariates that we have already adjusted for, they cannot have a risk ratio higher than 3.23 with both the maternal cancer and the outcome. Second, unlike previous studies, we could not exclude hereditary cancer syndromes, which may contribute to increased cancer risk in offspring [7, 8]. However, identifying hereditary cancer syndromes using this large population-based data is impractical. Moreover, hereditary cancer cases may constitute only a very small portion of the overall population. Third, cancer incidence in offspring is rare, leading to low statistical power and hindering comprehensive subgroup analysis (e.g., according to therapy). While the overall dataset provides meaningful results, the interpretation of strata defined by maternal age, cancer type, or the time interval between cancer diagnosis and pregnancy must be approached with caution. Fourth, we included only the first offspring born to cancer survivors to facilitate a clear comparison between the two groups and ensure a sufficient follow-up period, which limits generalizability.

## Conclusions

In conclusion, our study indicated that offspring born to cancer survivors had an increased risk of cancer, encompassing all subgroups of maternal age at delivery, maternal age at cancer diagnosis, and the interval between maternal cancer diagnosis and pregnancy. Further research on the long-term outcomes of offspring born to cancer survivors, such as cancer risk, is warranted to provide comprehensive cancer survivorship care.

## Supplementary Information

Below is the link to the electronic supplementary material.

Supplementary file1 (DOCX 22 KB)

## Data Availability

The data used in this study was approved by the Korean National Health Insurance Service The data underlying this study are sourced from the Korean National Health Insurance Service (K-NHIS) database, which covers approximately 99% of the South Korean population. Due to data privacy regulations, the data are not publicly available. However, researchers can request access to the K-NHIS database through the National Health Insurance Data Sharing Service, subject to approval. Further details on data access policies and application procedures can be found at K-NHIS Data Sharing Service (https://nhiss.nhis.or.kr).

## Abbreviations

K-NHIS: Korean National Health Insurance Service
ICD: International Statistical Classification of Diseases
PS: Propensity score
SMD: Standardized mean differences
NHL: Non-Hodgkin lymphoma
HR: Hazard ratios
CI: Confidence intervals
SIR: Standardized incidence ratio
IRB: Institutional Review Board
