# Supplementary material for: Association between maternal cancer and the incidence of cancer in offspring
Source: Eur J Epidemiol. 2025 Feb 17;40(2):177–85. doi: 10.1007/s10654-025-01206-z (PMC12018610; doi:10.1007/s10654-025-01206-z)
Supplement: Supplementary file 1 — Supplementary file1 (DOCX 22 KB) [file 10654_2025_1206_MOESM1_ESM.docx]

**Supplementary Appendix**

**Supplement to: Association between maternal cancer and the incidence of cancer in offspring**

**Supplementary Table 1.** Target Trial Specification for causal inference

| **Protocol Component** | **Target Trial Specification** | **Emulation Using Observational Data** |
| --- | --- | --- |
| Eligibility criteria | - All live births - Children born to females under 40 years of age who gave birth to their first child. | Same as for the target trial. Our cohort included all live births between January 1, 2005, and December 31, 2019, with a washout period of 2004 and a follow-up period of 2020.  Among them we selected children born to females under 40 years of age who gave birth to their first child.  By including all live births from a national dataset, we reduced selection bias, ensuring a representative sample of the population. |
| Exposure strategies | 1) The mother had history of cancer at birth  2) The mother without cancer at birth | Same as the target study.  1) Mother had a history of cancer at birth, defined by ICD-10 codes (C) or cancer-specific code (V193) prior to birth. However, due to concerns about overdiagnosis of thyroid cancer in Korea, we excluded thyroid cancer as a definition.  2) Mother without cancer at birth |
| Exposure assignment | Children are randomly assigned to mother with or without history of cancer at birth | Children were nonrandomly assigned to mother with or without history of cancer at birth. Randomization was emulated using propensity score matching adjusting for baseline covariates |
| Outcomes | Primary outcome: Cancer incidence among offspring | Same as for the target trial  We defined cancer incidence using the cancer-specific claim code.  All cancer patients are registered in the National Cancer Registry with a specific code (V193) to receive special insurance benefits in Korea. Thus, cancer is reliable in Korean claims data. |
| Follow-up | Follow-up began from birth and continued until cancer diagnosis, death, or end of the study period | Same as for the target trial.  Since our dataset comprehensively covers health care utilization, we expect minimal missing data. |
| Causal contrasts | Intention-to-treat effect | Observational analogue of intention-to-treat effect |
| Statistical analysis | Intention-to-treat analysis  Subgroup analyses by maternal age at delivery.  The cancer group was stratified by maternal age at cancer diagnosis, maternal cancer type, and time from maternal cancer diagnosis to pregnancy. | Same as for the target trial with adjustment for baseline covariates  Same subgroup analyses |

**Supplement table 2. ICD-10 codes to define the type of cancer**

| **Cancer type** | **ICD-10** |
| --- | --- |
| NHL | C82 - C86, C96 |
| Brain | C70 - C72 |
| Breast | C50 |
| Colon-rectal | C18 - C20 |
| Head and neck | C00 – C14 |
| Hodgkin lymphoma | C81 |
| Kidney | C64 |
| Leukemia | C91 - C95 |
| Liver | C22 |
| Testis | C62 |
| Thyroid | C73 |
| Stomach | C16 |
| Gallbladder | C23, C24 |
| Pancreas | C25 |
| Larynx | C32 |
| Lung | C33, C34 |
| Cervix | C53 |
| Uterus | C54 |
| Ovary | C56 |
| Prostate | C61 |
| Bladder | C67 |
| Multiple myeloma | C90 |
| Others | Other C |

**Supplement table 3. Age at diagnosis and cancer type among offspring.**

|  |  | **Maternal cancer** | |
| --- | --- | --- | --- |
|  | **Overall**  **(N=49)** | **Non-cancer**  **(N=30)** | **Cancer**  **(N=19)** |
| **Age at diagnosis, years (mean (SD))** | 2.0 (1.5) | 2.1 (1.6) | 1.9 (1.4) |
| **Age at diagnosis, years (median (IQR))** | 1.7 (0.8, 3.3) | 1.9 (0.7, 3.5) | 1.4 (1.0, 3.2) |
| **Cancer type** |  |  |  |
| Leukemia | 13 (26.5) | 8 (26.7) | 5 (26.3) |
| Liver | 5 (10.2) | 3 (10) | 2 (10.5) |
| Brain | 4 (8.2) | 1 (3.3) | 3 (15.8) |
| Non-Hodgkin lymphoma | 3 (6.1) | 2 (6.7) | 1 (5.3) |
| Testis | 1 (2.0) | 1 (3.3) | 0 (0) |
| Head and neck | 1 (2.0) | 1 (3.3) | 0 (0) |
| Thyroid | 1 (2.0) | 0 (0) | 1 (5.3) |
| Others | 21 (42.9) | 14 (46.7) | 7 (36.8) |

Values are expressed as N(%).
